# The processing of familiar English L2 phrasal verbs in neutral and biased sentence contexts

**DOI:** 10.3389/fpsyg.2025.1528821

**Published:** 2025-06-05

**Authors:** Jianyao Yu, Siya Wang, Ping Zhang, Tai’an Chen

**Affiliations:** ^1^School of Foreign Studies, South China Normal University, Guangzhou, China; ^2^Guangdong Center for Language Cognition and Assessment, Guangzhou, China; ^3^Cheng Yi Middle School, Xiamen, China; ^4^School of Foreign Languages, Soochow University, Suzhou, China; ^5^Maoming School Affiliated to Guangdong Experimental High School, Maoming, China

**Keywords:** familiar phrasal verbs, eye-tracking, preferential meaning activation, context effect, proficiency effect

## Abstract

This paper addresses an important psycholinguistic question: whether L2 learners preferentially process phrasal verbs (PVs) literally or figuratively, irrespective of context and proficiency levels. Our primary aim was twofold: first, to investigate how familiar English L2 PVs are processed—whether literally or figuratively—and secondly, to explore this across different contexts (neutral, literal bias, figurative bias) and proficiency levels among learners. Drawing on existing literature, we tentatively hypothesized that while learners might activate literal meanings early in processing, figurative activation could dominate later stages as far as familiar PVs are concerned. Familiarity with PVs may be critical across proficiency levels in driving PV processing. What’s more, the preferred meaning may be bootstrapped in supporting context, but the less preferred meaning is likely suppressed even with context boost. To achieve these objectives, an eye-tracking experiment was conducted with intermediate and advanced Chinese English L2 learners. Participants read context sentences containing PVs followed by a visual word search task to assess PV meaning activation at early, late and further delayed stages. Statistic analysis revealed that no consistent interpretation of PVs as literal or figurative in the time course emerged. However, in sentence reading, we observed faster late meaning activation in both literal and figurative contexts than in the neutral context, and delayed preference for figurative interpretation in the post-PV region. Meanwhile, in visual word search task, meaning activation was context-dependent and fluctuated over time, indicating temporal dynamics in processing. Last but not least, proficiency ranging from intermediate to advanced levels did not significantly impact processing when PV familiarity was achieved. Our findings suggest that teaching strategies should focus on enhancing learners’ ability to recognize figurative meanings. This approach could improve reading comprehension by promoting learner awareness of PVs as whole lexical units. In conclusion, this study enhances our understanding the mechanisms underlying L2 PV processing dynamics and provides actionable insights for language acquisition strategies, thus contributing valuable knowledge to the field of second language processing and learning.

## Introduction

Recent decades of psycholinguistic research has underscored the importance of focusing on multiword unit processing and acquisition (Arnon and Snider, 2010; Jiang and Siyanova-Chanturia, 2023; Jolsvai et al., 2020; Wray, 2002). As one type of multiword unit, phrasal verbs (PVs) have been under-researched due to their complex nature with regard to their syntactic and semantic aspects (Wisintainer and Mota, 2018).

PVs abound in the English language (Gardner and Davies, 2007) and challenge L2 English learners because of their complex nature (Side, 1990). Previous research focused more on L2 PV learning, learner difficulty in using PVs and their avoidance behavior (e.g., Dagut and Laufer, 1985; Liao and Fukuya, 2004; Siyanova-Chanturia and Schmitt, 2007; Zhang and Chen, 2019). What remains underexplored about PVs is how L2 learners process PVs, which is important to facilitate our understanding of whether L2 learners employ similar underlying cognitive mechanism in processing PVs as in dealing with idioms (typical multiword units). Increasingly more research supports treating idioms both figuratively as holistic lexical units and literally as strings of individual words in figurative language comprehension (Yi and Zhong, 2023). We join in this theoretic discussion by testing this dual approach on PVs.

In this study, we aim to conceptually replicate Wang et al. (2016), investigating how intermediate and advanced Chinese learners of English process familiar L2 phrasal verb (PV) constructions in neutral, literally biased, and figuratively biased sentence contexts. Our investigation is two-staged: tracking eye movements in both PV and post-PV noun phrase regions in sentence reading, and following it with a visual word search task to examine further delayed PV processing effects. The findings from this study could provide insights into improving PV comprehension by helping learners understand the importance of knowing opaque figurative meanings.

## Theoretical background

### Figurative language processing models and related influencing factors

The study of figurative language processing has been marked by the debate surrounding how figurative and literal meanings are processed during idiom comprehension. Currently the more widely accepted approach to figurative language processing is the hybrid model. According to this view, idioms are processed as both single lexical units and phrases composed of individual words, with both figurative and literal meanings being activated simultaneously (Beck and Weber, 2016; Yi and Zhong, 2023). However, the activation of these meanings is not necessarily sequential, beginning with literal interpretation. The graded salience hypothesis in this approach proposes that it’s a graded process where more salient meanings (e.g., conventional, frequent, familiar) are preferentially processed over less salient ones (Giora, 1997, 1999, 2002). In accordance with graded salience hypothesis, familiar meanings - whether literal or figurative - are more accessible and salient for speakers/hearers. This means that figurative meanings can be easily accessed for familiar idioms, while literal meanings serve as the default interpretation for unfamiliar ones.

Building upon the graded salience hypothesis, Cieślicka (2006) put forward the literal salience resonant hypothesis which offers a unique perspective on L2 idiom processing. Specifically designed to account for comprehension of L2 idioms, it predicts that L2 learners tend to process L2 idioms literally, regardless of context and familiarity. L2 learners supposedly become familiar with individual words before encountering the idioms themselves, which affords literal meanings a more salient status. Empiracal studies from Cieslicka and colleagues supported this hypothesis by showing that L2 learners prefer literal interpretations for idioms (Cieslicka, 2011; Cieslicka and Heredia, 2011, 2019; Cieslicka et al., 2014).

However, more recent research on the hybrid approach to idiom processing by both L1 and L2 speakers has challenged this hypothesis. For instance, studies have shown that speakers can both access/retrieve figurative meanings and analyze individual words (e.g., Holsinger, 2013; Kessler et al., 2020; van Ginkel and Dijkstra, 2020). This raises questions about the extension of literal salience resonant hypothesis to L2 learners’ processing of idioms and phrasal verbs.

Other research illuminates more complexities involved in idiom processing by focusing more on the factors influencing literal or figurative interpretation. Familiarity (subjective frequency) has been deemed as the primary predictor of figurative interpretation of idioms and also an index of ease of direct retrieval of figurative meanings (Carrol and Littlemore, 2020; Libben and Titone, 2008; Schweigert and Moates, 1988; Titone and Libben, 2014). Studies by Libben and Titone (2008) and Titone and Connine (1999), among others, have highlighted the impact of familiarity with idioms on meaningfulness judgments. Participants are more likely to process an idiom as figurative if they possess knowledge about its intended non-literal interpretation. One cannot expect participants to consider the figurative meaning of an expression if they do not know the said meaning (Holsinger, 2013). Schweigert and Moates (1988) suggest that without idiom knowledge, participants may default to literal interpretations regardless of their proficiency level. Kim (2016) emphasizes that when dealing with less well-known or unfamiliar idioms, even proficient L2 learners may misconstrue such idioms as literal due to their deceptive transparency. Such findings highlight the necessity to include tasks to ascertain the actual familiarity with the figurative language in question before assessing whether literal or figurative meanings of idioms are processed preferentially.

In addition to familiarity, other factors such as transparency/compositionality and predictability also influence idiom processing (Libben and Titone, 2008; Titone and Connine, 1999). These factors may interact in modulating idiom processing at different time points. For example, transparency/decomposability only influences meaningfulness judgments if idioms are less familiar (Libben and Titone, 2008). Idiom literality also plays a role in idiom processing. Idiom recognition may come earlier with greater idiom familiarity but later with higher literality of an idiom (Morid et al., 2021; Titone and Libben, 2014). Furthermore, context weighs in the process by either promoting or suppressing figurative interpretation. For instance, there is speedy figurative processing of high-familiarity idioms in figuratively biased contexts and literal processing of low-familiarity idioms in literally biased contexts (Wang et al., 2021). There is also interaction between biasing context and idiom literality. For example, supporting literal or figurative context strengthens the biased meaning interpretation for high-literality idioms (e.g., “*at the end of the day*”), but there is a cost of incongruent meaning resolution; meanwhile, for low-literality idioms (e.g., “*in the seventh heaven*”), literal interpretation carries a cost regardless of literal or figurative context (Beck and Weber, 2020). To sum up, such factors as familiarity, transparency, and context bias should all be considered in further research into L1 figurative language processing.

Research has shown that the above factors also influence L2 idiom processing. Familiarity plays a critical role in determining how learners process idioms (Zhou and Zhang, 2011). Titone et al. (2015) study found that both direct retrieval and compositional analysis are involved in L2 idiom processing. In particularZhou and Zhang (2011) experiment demonstrated the importance of familiarity with L2 idioms. They presented Chinese learners of English with neutral or figuratively biased contexts with idioms embedded, then the participants were required to fill in literal and figurative associate words. The results showed that when processing familiar idioms, both higher-level and lower-level learners preferred figurative meaning interpretation of the idioms in both neutral and figuratively biased contexts. In contrast, when processing unknown idioms, the proficiency effect emerged: While both levels of learners favored literal meanings in neutral contexts, they differed in their interpretation of idioms in figuratively biased contexts. Higher-level learners opted for figurative meanings, whereas lower-level learners preferred literal meanings (Zhou and Zhang, 2011). These findings contradict Cieslicka’s hypothesis that L2 speakers always prefer the literal meaning and instead support Giora’s graded salience hypothesis (Giora, 1999) by evidencing familiarity effect.

In short, the analyses of the influencing factors on figurative language processing provide new insights for further study to uncover the underlying processes involved in PV interpretation in context across varying L2 proficiency levels.

### Previous research on phrasal verb processing

Phrasal verbs (PVs) are verb particle combinations syntacally (Cappelle et al., 2010; Richards and Schmidt, 2011). Semantically, PVs have been viewed as lexical units along a continuum (Dagut and Laufer, 1985; Darwin and Gray, 1999; Dixon, 1982; Palmer, 1974) with PVs like “*walk in*” (whose meaning is transparent) and “*give up*” (which has an opaque figurative sense only) at the two ends of this continuum. In terms of Cappelle et al. (2010), the PVs at the two opposite ends are literal and figurative PVs. Many PVs along this continuum have figurative meanings and are also literally plausible (of high litreality), such as “*run into [encounter] someone*” or “*run into [enter] a room*.” To test whether L2 learners process PVs literally or figuratively, this study focuses on these PVs which are both literally and figuratively plausible (e.g., *run into*).

Researchers have considered idiom processing models applicable to L2 PV processing (e.g., Carrol and Conklin, 2020; Dagut and Laufer, 1985; Siyanova-Chanturia and Martinez, 2014), given the formulaic similarities between idioms and PVs (Wray, 2002). However, studies on PV processing are limited in number. Preference for figurative processing of PVs was found with L2 learners in several studies (Blais and Gonnermn, 2013; Matlock and Heredia, 2002; Paulmann et al., 2015). For instance, Matlock and Heredia’s study (2002) found that advanced early bilinguals processed figurative meanings for PVs similarly to native speakers, whereas lower-level late bilinguals relied more heavily on literal interpretations. This suggests that L2 proficiency may influence the way learners process PVs. The study by Matlock and Heredia provides valuable insights into how L2 learners process PVs, but it has several limitations that can be addressed in future research. One limitation is the reliance on self-paced reading tasks on the sentence level, which may not provide precise information about how learners engage with the PV within a sentence (Blais and Gonnermn, 2013). Another limitation is the lack of control for PV familiarity among participants, as prior exposure to a PV could influence processing. Additionally, the study did not account for PV transparency levels or syntactical complexity of sentences where PVs were embedded, which may impact figurative or literal interpretations. Furthermore, contextual cues such as sentence topic and surrounding discourse were not controlled for, which could also affect learners’ preference for figurative interpretation.

The study by Paulmann et al. (2015) also found that both upper-intermediate-to-advanced L2 learner group and native L1 speakers showed similar preference for figurative meanings when reading PVs, such as “*run over*” meaning *to kill someone*. This was evident from the event-related potential (ERP) data, which indicated smaller N400 component for the following noun phrase allowing for figurative meaning (e.g., *the old man*) than the one allowing for literal meaning (e.g., *the old bridge*). However, there are some limitations to this study that should be considered: Firstly, the study only included one group of L2 learners compared to a native speaker group, leaving it unclear whether these findings would generalize to lower-proficiency L2 learners. Future research could benefit from including different proficiency levels to examine potential differences in idiomatic interpretation. Secondly, the study only examined the ERP components for paired nouns after reading the same PV. Future research could benefit from examining the effect of prior literally or figuratively biasing context on the same PV plus the same noun phrase (e.g., *Peter was looking around for Black Friday deals. So Peter ran into Zara on Oxford Street* vs. *Peter had not seen Zara for years. Then Peter ran into Zara on Oxford Street* in which Zara is likely to be interpreted as a chain store in one situation or a friend in the other situation). This would allow researchers to better isolate the effect of prior biasing context and gain insights into how L2 learners process PVs in different contexts.

Evidence from Blais and Gonnermn (2013) suggests that advanced L2 speakers may process phrasal verbs in a manner similar to native speakers. In their study, researchers used a masked priming task to explore automatic activation patterns for figurative meanings in L2 speakers’ mental lexicon. The results showed that high-proficiency L2 speakers exhibited response latencies similar to those of native speakers when processing transparent and opaque phrasal verbs. Specifically, the study found that transparent PVs (e.g., “finish up”) produced greater priming on their component verbs (e.g., “finish”) than opaque PVs (e.g., “chew out”) did on theirs (e.g., “chew”). This suggests that L2 speakers with higher proficiency are sensitive to the figurative phrasal verbs, which can cause a delay in processing. In contrast, lower-proficiency L2 learners showed no response difference between transparent and opaque PVs. The study’s findings suggest that L2 proficiency plays a role in phrasal verb processing. However, it is essential to note that this experiment only tapped into the priming effect of different types of PVs on their component verbs out of context. Additionally, similar to Paulmann et al. (2015) research, the study did not assess learners’ familiarity with PVs’ opaque meanings at either proficiency level.

In contrast to the studies in favor of preferential figurative processing, other PV processing studies support opposite primary literal activation in both L1 and L2. Holsinger and Kaiser (2013) found that native speakers tend toward default literal processing of phrasal verbs. In their self-paced reading task, participants were presented with ambiguous PVs that could be interpreted in either a literal or idiomatic sense (e.g., *dig into the tomb/sandwich*). The study’s design relied on creating semantic contradictions between the prior biasing context and the disambiguation context following the PV (e.g., *The daring archaeologist/hungry waitress who had been working all day dug into the sandwich/tomb just after noon on Sunday.*). The findings suggest that native speakers may prioritize literal meanings over figurative ones, which could be more attributed to the complex contextual semantic contradiction. It is unclear whether this tendency would also apply to L2 learners, who might have uneven level of familiarity with idiomatic senses of PVs. Additionally, it’s worth noting that Holsinger and Kaiser (2013) did not control for PV transparency, which could have impacted their findings.

Wang et al. (2016) study with L2 learners further supports the idea that phrasal verbs may be processed primarily literally. In their visual word search eye-tracking experiment, intermediate and advanced English L2 learner groups were presented with either literally or figuratively biased context sentences containing a PV in each sentence, and then had to choose one of the four probe words (literally or figuratively related words, and two unrelated control words). The results from the eye fixation data on the probe words showed an overall reliance on literal meaning activation at both proficiency levels in the task. Besides, L2 learners spent more time considering context-biased literal or figurative meanings, and there was also an L2 proficiency effect in the activation of figurative meanings in the literally biased context, with only the advanced group showing figurative activation in spite of the literal bias. The strength of this study is its control for familiarity with both literal and figurative meanings, which was lacking in previous PV studies. However, the use of visual word search eye-tracking measures only allowed the researchers to examine L2 learners’ delayed cognitive processes of PV interpretation, which they confused for online PV processing. Besides, it’s also worth noting that there was no neutral baseline context for comparison and the total fixation time proportion analysis used in Wang *et al.*’s study may not have captured finer-grained temporal dynamics as demonstrated in Holsinger (2013). This limitation highlights the importance of updating statistical analytical approaches when examining language processing patterns (see the next Section below).

To sum up, the current status quo of phrasal verb (PV) processing research presents several unanswered questions. For instance, do all non-native English speakers process PVs literally? Do they tend to interpret PVs figuratively or literally in contexts that are biased toward literal or figurative meanings? Additionally, is there a consistent pattern in the time course of meaning processing? The discrepancies between studies such as Wang et al. (2016) and Paulmann et al. (2015) highlight the complexity of how non-native English speakers process PVs figuratively versus literally. While the different studies have examined individual factors like transparency, context biasing, level of familiarity or language proficiency in isolation, future research should aim to consider these variables simultaneously within a single experimental framework in light of the findings about the complexities involved in idiomatic/figurative language processing (See the previous Section). To achieve this multifaceted understanding, researchers could manipulate contextual biases and examine how prior contexts influence subsequent PV interpretations. Moreover, it is essential to control for the figurative meaning familiarity of target PVs, as previous studies have shown that non-native speakers tend to misconstrue less well-known idiomatic expressions as literal due to their deceptive transparency (Kim, 2016). Furthermore, given the intertwined nature of familiarity and proficiency effects in idiom comprehension (Zhou and Zhang, 2011), future research should strive to disentangle these joint effects on non-native speakers’ processing of PVs. By addressing these gaps in our understanding, we can contribute to developing models of phrasal verb processing across L2 proficiency levels.

### Related research using visual word search eye-tracking paradigm

Eye-tracking allows us to gain insights into the real-time consideration of literal and idiomatic interpretations over the time course of sentence comprehension without the need for predetermined time-point selections. The visual word search (VWS) paradigm in particular has been adapted to investigate the processing of figurative language, such as idioms and phrasal verbs. By measuring gaze patterns across different regions of interest on the display, this method captures the temporal dynamics of comprehension through changes in eye movement focus. Operationally, participants’ consideration of literal or figurative meaning in VWS is assessed by analyzing the proportion of eye fixations directed toward literal versus figurative related probe words compared to their respective distractor words. Higher proportions of looks indicate a greater degree of attention to and consideration of that particular meaning.

The listen-and-look visual word search paradigm involves listening to spoken information while viewing visual probe words on display (McQueen and Viebahn, 2007). A read-only variant of the paradigm is to read a sentence before viewing the visual probe words (Holsinger, 2013). By examining how participants’ eyes move between semantically related words, and unrelated control words in the same grid, we can gain insights into whether individuals are relying more heavily on lexical recognition of PVs or performing word-by-word syntactic analysis.

Observable eye movements in response to a target word are often within the 1,000-ms time window from the target word onset (Allopenna et al., 1998; Yee and Sedivy, 2006). Moreover, according to Ito and Knoeferle (2023), a more rigorous approach to statistical analysis is to count fixations in very fine time bins (e.g., 20-, 50- or 100-ms interest period), then partition the bins into wider time windows (e.g., 400 ms) and observe the looks proportion in each time window. Moreover, for interest period (time bins) shorter than 100 ms, eye fixation “yes/no” data are binomially distributed and can be transformed into binary (1, 0) data. Analyzing binary gaze data at multiple time points (for example in 20-ms bins), rather than relying on single measures such as fixation durations or total numbers of fixations helps to account for heteroscedasticity (variation in the variance of fixation durations), improve the sensitivity of statistical analysis and reduce Type II errors. However potential problems with multiple testing in 20-ms bins could lead to an increase in false significant results. To reduce the likelihood of spurious significance, eye movement data should be averaged over larger time windows (e.g., of 400 ms) (Ito and Knoeferle, 2023).

It was along these lines that Holsinger (2013) converted gaze sampling data in his listen-and-look visual word search eye tracking task into binary data and divided them between early and late time windows (180–580 ms,^1^ 580–980 ms). He investigated how native speakers process idioms in neutral, literally-biased, and figuratively-biased contexts (e.g., *kicked the bucket*), obtaining the fixation count proportions and the order in which the native speaker participants fixated on a set of four visual probe words presented simultaneously with the onset of the auditory idiom embedded in sentence. His findings showed that, in unbiased conditions, participants initially preferred looking at literal associates before experiencing attention competition between figurative and literal associate words later on. In literally biased contexts, looks to literal associates were significantly higher than looks to unrelated control words in both time windows, while in figuratively biased contexts there was only early consideration of literal meanings as measured by higher looks proportions. The time-course statistical analysis of L1 idiomatic processing mechanisms provides a useful frame of reference for our study of L2 PV processing.

In contrast to Holsinger (2013) study, the experimental paradigm adopted by Wang et al. (2016) was a read-only visual word search variant (see the previous section), but relied only on the proportions of total interest area reading/dwell time for the probe words (e.g., 4,000 ms, much longer than the usual 1,000-ms observation window) to make statistical analysis, and were unable to account for the heteroscedasticity of the total reading time. Another limitation is that they did not provide information on early online processing of phrasal verbs but instead relied solely on total reading time analysis for probe words. In this approach they quickly drew an unsupported conclusion about early activation of literal meanings for the phrasal verbs at the earlier stage of sentence reading. Therefore, to further examine the time-course changes in meaning activation, it is necessary to observe not only the participants’ fixations to probe words, but also fixation data during earlier sentence reading.

Further research should aim to improve upon these limitations by focusing on the timing and order of literal and figurative meaning consideration while employing more robust statistical analyses. By doing so, researchers can gain a better understanding of nuanced temporal patterns in PV processing by L2 learners of different proficiency levels.

## The present study

The present study replicates Wang et al. (2016) by employing the same read-only visual word search paradigm but followed the statistical analysis approach in Holsinger (2013). Specifically, this study investigates how Chinese learners of English at intermediate and advanced proficiency levels process familiar PVs with similarly high literal and figurative meaning familiarity ratings but significantly different literal and figurative transparency ratings. The goal is to examine whether distinct proficiency groups of L2 learners give PVs a literal interpretation in preference to a figurative interpretation in literally biased, figuratively biased, and neutral unbiasing prior contexts.

### Research questions

Based on our review of previous studies, we address two research questions as follows:

1. Do learners show continuous preference for activating either literal or figurative meanings across different contexts at both early sentence reading stage and late visual probe word search stage when PVs have equivalent familiarity ratings?
2. Does L2 English proficiency affect the processing order or activation strength in interpreting PV meanings at both early sentence reading stage and late visual probe word search stage when PVs have equivalent familiarity ratings?

Competing meaning activation models present us a challenging task to predict either literal or figurative meaning activation in figurative language processing. Giora’s graded salience hypothesis, when applied to L2 learners who are familiar with figurative language, suggests that they should activate meanings figuratively regardless of context. However, context itself, as a driving factor in achieving salience effect, is known to influence whether interpretations lean toward literal or figurative meanings based on the context provided. Empirical studies indicate that even highly competent L1 speakers may find themselves activating literal meanings for familiar figurative language in figuratively biased contexts during early stages of online processing.

According to the literal salience resonant model, which applies specifically to L2 learners, literal meaning should be superior and preferred over figurative meaning when processing a familiar PV regardless of context. This implies that literal interpretations should take precedence irrespective of how a PV is presented or used. For bilinguals with a less proficicent L2 at least, literal meanings are more salient and therefore literal activation should be obligatory throughout the time-course of processing as shown in lexical decision tasks immediately at sentence offset, 300 ms, or 800 ms after sentence offset (Cieslicka and Heredia, 2011). Nonetheless, research by Paulmann et al. (2015), along with other studies, reveals that proficient L2 learners may exhibit similarities to native speakers in their default preference for early figurative processing. These findings suggest a more nuanced understanding instead of an absolute either/or choice between literal and figurative meanings during activation.

Therefore, we tentatively suggest that L2 learners, in dealing with familiar PVs, may initially activate the literal meaning like L1 speakers before preferred figurative interpretations become dominant as online processing continues. Additionally, we also expect that context may bootstrap the preferred meaning earlier and more strongly than the less preferred meaning.

The role of L2 proficiency in processing figurative vocabulary (PVs) is less straightforward than previously thought. Previous studies suggest that only advanced learners exhibit a figurative advantage when interpreting PVs. However, when item familiarity is controlled for, even lower-proficiency learners tend to activate figurative meanings of highly familiar idioms during explicit tasks like blank-filling semantically related words. These findings challenge the literal salience resonant hypothesis by revealing that lower proficiency learners still prefer figurative interpretations of familiar idioms. This suggests that intermediate and advanced L2 learners may exhibit similar tendencies when processing such idioms, even if their proficiency levels vary. Further research is needed to determine whether this figurative preference holds for PVs over time during real-time interpretation in sentence-level reading and succeeding probe word reading.

The previous research pointed to two opposite directions. Therefore, we did not have a definitive hypothesis. We explored whether it would happen as we tentatively suggested. Statistically, Hypothesis_0_ was: there would be no meaning activation preference for target items (PVs, post-PV noun phrases, or probe words) across contexts and proficiency levels. The alternative hypothesis was: there would be meaning activation preference across contexts and proficiency levels.

## Experiment method

### Participants

A statistical power analysis based on a pilot test with 27 participants homogeneous to students in the main experiment and the same materials as in the main experiment showed that to obtain the sufficient statistical power of 0.8, we should have a sample size of 80 participants.

Participants were recruited from South China Normal University, with all being native Chinese speakers who had no prior experience studying or living in English-speaking countries. A total of 99 students participated in the main experiment, receiving a payment of 40 RMB each as an incentive for their involvement.

The intermediate group comprised 51 sophomore students majoring in English (who were pre-TEM-4 but all passed TEM-4^2^ the next semester). The advanced group consisted of 48 English graduate students who had already passed TEM-8. A significant age difference was found between proficiency groups (*M*_*intermediat*e_ = 19.44, *SD* = 0.87, *M_advanced_* = 23.17, *SD* = 1.19; *t* = 17.13, *p* < 0.0001, *Cohen’s d* = 3.56).

To ensure a clear distinction between intermediate and advanced levels, we excluded students who had exceptionally high scores on TEM-4 (above 90 out of 100). Participants’ vocabulary sizes were measured using the Vocabulary Size Test (VST) (Nation and Beglar, 2007) as vocabulary knowledge has often been used to indicate proficiency in studies on L2 formulaic language processing (e.g., Milton, 2010; Sonbul et al., 2020; Wolter and Yamashita, 2018). The results showed a significant difference between groups in terms of vocabulary size (*M_intermediate_* = 56.29, *SD* = 8.59, *M_advanced_* = 88.13, *SD* = 14.12; *t* = 13.29, *p* < 0.0001, *Cohen’s d* = 2.70).

### Research materials

The research materials included PVs embedded in context sentences and probe words for visual display.

### Item selection

To ensure that even the intermediate participants would be familiar with the PVs used in this study, we started by identifying potential PVs from the TEM-4 syllabus and then screened them based on semantic transparency, transitivity, and COCA frequency. This process eliminated PVs that were not suitable for our purposes.

To confirm the idiomatic/figurative meanings of these PVs, we consulted the Longman Phrasal Verbs Dictionary (Pearson Education, 2000) and checked their literal plausibility in COCA. We also used a stratified sampling method to examine the percentages of selected literal and figurative meanings in a sample of 100 lines for each PV. This process allowed us to exclude two PVs with minimal frequency.

We then invited 24 Chinese sophomore English majors (at the intermediate level) to write out in Chinese the first two meanings that came to mind when they saw each PV. Based on their responses, six PVs whose figurative meanings did not come up for more than half of the participants were removed.

We also asked the same 24 sophomore English majors to rate the familiarity of both literal and figurative meanings on a five-point Likert scale (see Wang and Koda, 2005; Zhang and Wen, 2019). Six PVs with average familiarity scores below three for their figurative meanings were excluded. The remaining final 13 PVs showed no significant difference in literal and figurative meaning familiarity ratings (*M_literal meaning_* = 4.06, *SD* = 0.58 and *M_figurative meaning_* = 4.06, *SD* = 0.48, *t* (12) = 0.03, *p* = 0.97, *Cohen’s d* = 0.01).

To prevent paired items like “dig into the tomb/sandwich” from contaminating the phrases pool, we carefully selected PVs that did not have obvious metaphoric extensions of their literal meanings. For example, we avoided using verb particles with close meaning similarities between the PV wholes and the component verbs (like “dig into”), which could blur the boundary between literal and figurative meanings.

To assess the semantic transparency of these PVs, we asked 24 advanced learners to rate the predictability of their literal and figurative meanings on a five-point scale (from completely unpredictable to completely predictable) (refer to Zhang and Wen, 2019). The results showed that the figurative meanings were significantly less predictable than the plausible literal meanings for each PV (*M_literal meaning_* = 4.48, *SD* = 0.28 and *M_figurative meaning_* = 3.21, *SD* = 0.37, *t* (12) = 11.45, *p* < 0.0001, *Cohen’s d* = 3.18). (refer to Supplementary material A for the selected PVs).

### Context sentences with literal or figurative bias

To ensure that the PVs were embedded in contexts that would bias interpretations, we designed three types of context sentences: literal, figurative, and neutral. We avoided separable PV structures (e.g., *put the picture up*) to minimize cognitive effort. Besides, we used the same noun phrase after the literal and figurative uses of the same PV [*ran into Zara* (a person) vs. *ran into Zara* (a shop)] so that we would see how the preceding context and the meaning salience of the PV itself would act on the same noun phrase and ensure that the processing difference would not come from the difference between the two noun phrases behind the same PV.

All target sentences (refer to Supplementary material B) followed a consistent pattern: “subject + PV + noun phrase + adverbial.” To ensure that our contexts were adequate for participants’ intended interpretations of the PVs, we pilot-tested five homogeneous advanced English learners who translated the biased sentences into Chinese. Sentences with 80% or more translations revealing the context-biased interpretation were retained. Neutral contexts were designed to be ambiguous and not to provide cues for either literal or figurative interpretations. Operationally, the same five people were first required to write down possible anticipanted meaning of the prior sentence and then compare the meaning with literal and figurative meanings of the target PV before deciding if the prior context cued neither meanings.

One example of embedding the same PV in different sentence contexts is shown below.

- Literal context: When Eva saw the fire burning on the bed, she ran across the house for water.
- Figurative context: When Eva was watching birds with a telescope, she ran across the house with big windows.
- Neutral context: Who knew what would happen to Eva next. She ran across the house in excitement.

In this example, we see the PV *ran across* used literally in one context [A] and figuratively in another [B], while the neutral context [C] does not provide cues for either interpretation.

To prevent participants from identifying specific PVs or contexts, we rotated the sentences into three lists. Each list had all 13 PVs in sentences, but each participant read only one list with a unique combination of context conditions. Each list had all the three context conditions in rotation (four PVs in one condition, another four in another condition and the remaining five in the last condition).

To prevent the participants from identifying the purpose of this study, 26 filler sentences were used to mix with the target sentences. We had 13 sentences with idioms, e.g.*, Anna had a cold, so she was feeling under the weather these days*, and 13 sentences with fixed collocations, e.g.*, After Ella had lived in the country, she could not get used to the city life*, as in the study by Paulmann et al. (2015). The filler sentences and experiment sentences were randomized to eliminate trial order effect.

### Probe words for visual display

To assess participants’ interpretation of PVs in the target sentences, we designed a set of four words for each PV to appear in each trial: one literal associate, one figurative associate, and two semantically unrelated control words. The positions of these probe words were fully counterbalanced across trials (Holsinger, 2013; Wang et al., 2016).

To ensure that the probe words did not influence participants’ responses through their frequency, orthographic complexity, or length, we matched the two associate words with the two control words on these characteristics (Cieślicka, 2006). There was only a small difference in word frequency between the four types of probes (*M_lit_related_* = 249,023, *M_fig_related_* = 143072.08, *M_lit_ctrl_* = 120767.69, *M_fig_ctrl_* = 116226.69; *p* = 0.73, *Partial η^2^* = 0.03, *Cohen’s f* = 0.16).

However, we noted a significant length difference among the probe words (*M_lit_related_* = 4.69, *M_fig_related_* = 6.46, *M_lit_ctrl_* = 4.62, *M_fig_ctrl_* = 6.15; *p* = 0.002, *Partial η^2^* = 0.27, *Cohen’s f* = 0.61). To account for this potential confound, we included word frequency and length as fixed-effect covariate factors in initial R modeling to ensure that these variables did not influence our results. (refer to Supplementary material A for the probe word sets).

**Figure 1:**
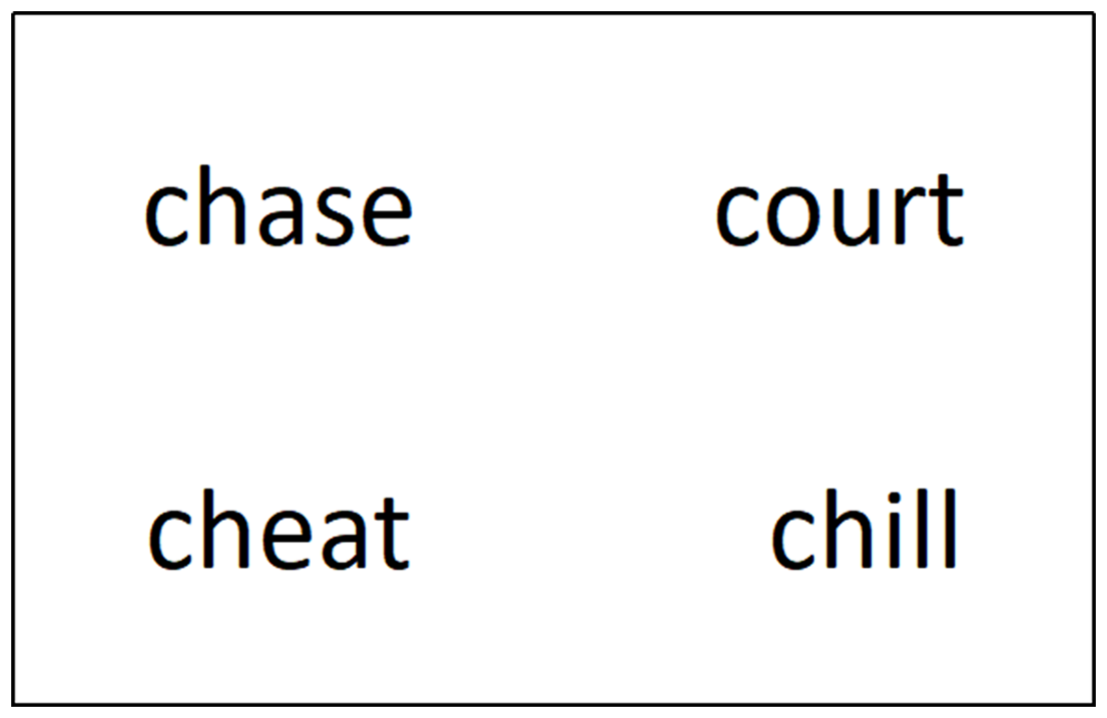
Sample visual display for the probe words of *run after*: The above left word *chase* is literally related, the above right word *court* is figuratively related, the below left word *cheat* is literal control word and the below right word *chill* is figurative control word. The positions of the four types of probe words are rotated in the visual word search task.

Here is an example of the visual display for the PV *run after* (Figure 1).

## Experiment procedure

The experiment stimuli were presented on a computer screen using Experiment Builder software (SR, 2021). The screen had a resolution of 1,024 × 768 pixels, with each region of interest in the four corners occupying approximately 369 × 248 pixels. Eye movements were tracked using an Eyelink 1,000 Plus tracker at a sampling rate of 1,000 Hz. Participants sat 65 cm from the screen and rested their chins on a chinrest to minimize head movement.

Before commencing the formal experiment, participants read instructions to ensure they understood the requirements. Eye movements were calibrated and validated to guarantee accurate data recording using a 9-point star calibration followed by five practice trials. If any participant’s eye movement deviated during the experiment, it was paused for recalibration and revalidation.

To minimize fatigue effects, participants had the option to request breaks before the next trial, which would be preceded by recalibration and revalidation of their eye tracker. The experiment began with a drift correction at the start of each trial when they focused on the fixation cross to the left of the sentence. Participants then read a context sentence presented in one line on the screen, which should reveal to us potential early and late meaning activation in PV processing. After completing the sentence, they clicked the mouse to proceed, followed by a 500-ms display of a red “?” question mark at the center of the screen. This was succeeded by the presentation of four probe words in the visual word search task. The visual word search task was designed to explore whether literal activation is also obligatory during two consecutive time windows at the delayed stage of processing after sentence offset as indicated in Cieslicka and Heredia (2011).

**Figure 2:**
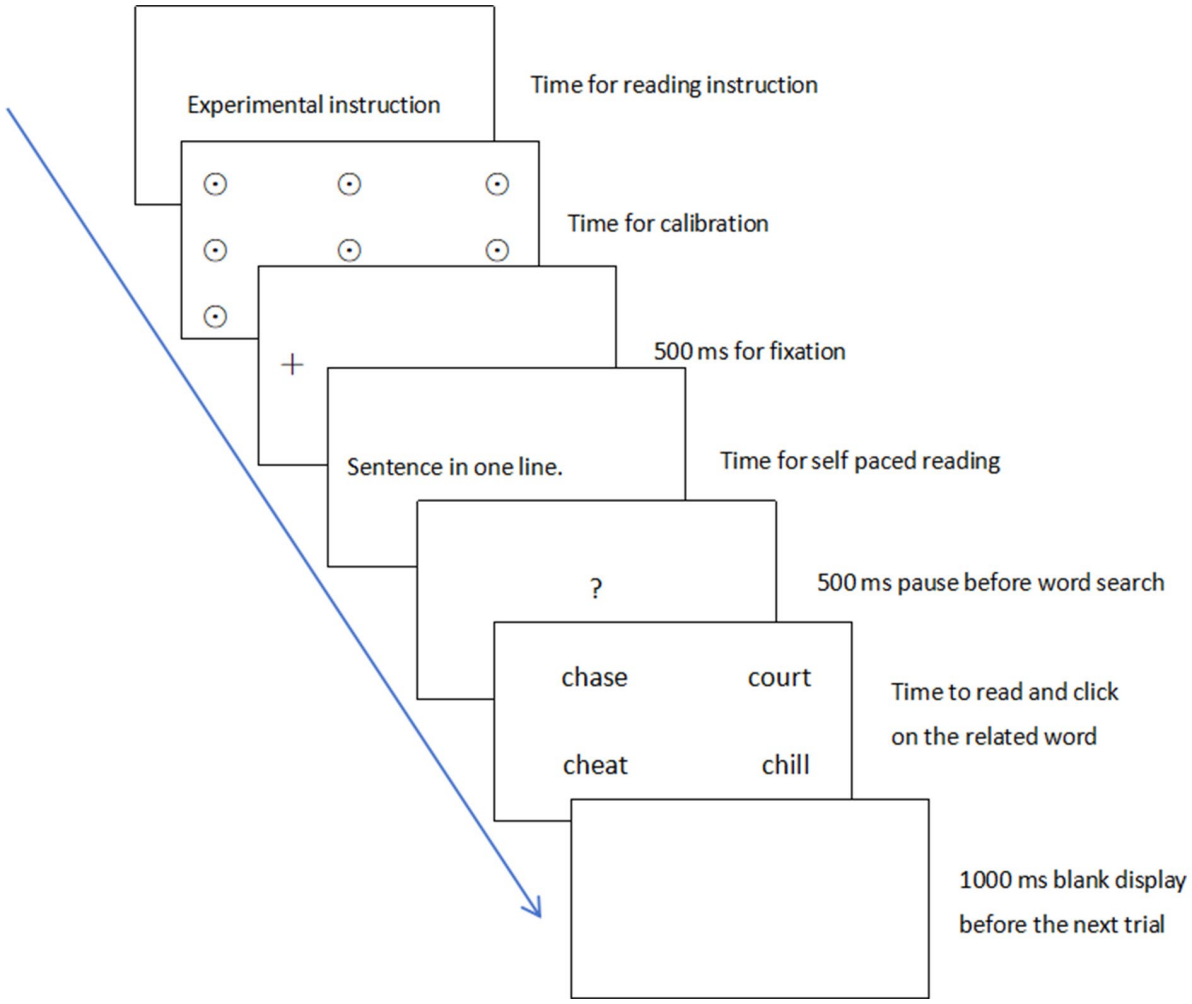
Eye movements experiment procedure.

Participants were instructed to make meaning-relatedness judgments as quickly and accurately as possible upon seeing the probes. They then clicked on their chosen word with the mouse. After each judgment, the screen went blank for 1,000 ms before the next trial began (see Figure 2). After the experiment, participants were surveyed to confirm their related PV meanings. Each participant rated each PV’s figurative meaning using their Chinese translation on a 5-point Likert scale from 1 (totally unfamiliar) to 5 (completely familiar). For example, the PV “*run after*” was presented together with its Chinese translation 追求 (“court (girls)”). Participants were asked to rate how familiar they felt with the figurative meaning of each PV. The survey results indicated that all data points were relevant and therefore were retained because they demonstrated sufficient familiarity with the figurative meanings of PVs. Ratings ranged from 4 to 5, with an average score of *M* = 4.48 and a standard deviation of *SD* = 0.37.

### Data preparation and analysis

Among the 99 participants, six finished the experiment task with the sampling rate accidentally set at 500 Hz, different from the normal 1,000 Hz. Data of another student was lost due to a computer hardware memory problem. Data from the other 92 students were valid for statistical analysis.

Regarding sentence reading, we concentrated early-measure and late-measure analyses on both PVs and the following noun phrases. Phrase-level effects may reflect how a construct is processed as a whole unit, while post-PV regions tend to show delayed processing effects (Carrol and Conklin, 2020). For early measures, we used *first fixation duration* (the duration of the first fixation on the focused phrase within a specific interest area) and *first pass reading time* (the duration of all the fixations on the phrase the first time it is encountered in the sentence before gaze exits to the left or right). These metrics capture initial processes like familiarity checks, access to orthographical/phonological information and lexical meaning, and early information integration. For late measures we adopted *total reading time* (also called total dwell time, the duration of all fixations on the phrase during the trial including time for rereading the same phrase) and *second pass reading time* (lookback fixation time) which refers to all the returning fixations within the interest area following the initial fixation (Hyönä et al., 2003). We chose those measures based on past literature (Reichle et al., 1998; Roberts and Siyanova-Chanturia, 2013). These two measures are believed to be sensitive to later processes associated with comprehension of a text, such as information re-analysis, recovery from processing difficulties and integration of information in discourse (Rayner et al., 1989; Paterson et al., 1999). Research indicates that significant late-measure effects without corresponding significant effects from early-measure indicators imply that the examined effects develop relatively late in the online processing (Yan et al., 2013).

To trim probe word reading data, we converted the looks to probes words in every 20-ms bin starting from 180 ms after the probe display onset into binary data and sorted them into two consecutive 400-ms time windows to prepare for inferential analysis. To do this, we aligned the eye-tracking data to the onset of the visual display for each set of probe words and counted the fixations on the probe words (looks) in each 20-ms time bin (interest time period). Since the Eyelink 1,000 Plus tracker samples at 1,000 Hz, there will be 1,000 samples of fixation on an area of interest if one focuses on the area for 1,000 ms. If there were more than ten samples out of 20 ones in a 20-ms time bin, it was coded one, otherwise it was zero. We converted the samples into binary data since for an interest period shorter than 100 ms, it is not likely that eyes move from one interest area to another. Fixation data within such a short time bin are likely to be binomially distributed and can be coded one for *fixated* or zero for *not fixated* (Ito and Knoeferle, 2023). The data was then sorted into two analysis windows: T1 window (180–580 ms) and T2 window (580–980) following the practice of Holsinger (2013). All probe word reading analyses were conducted on the binary data from the two windows.

We conducted our statistical analysis using R software (R Core Team, 2023), employing linear mixed-effects models from the lme4 package for analyzing PV and post-PV NP reading time data. For probe word reading responses—binary (fixation/no fixation) outcomes—we utilized generalized linear mixed-effects logistic regression models, following the methodology outlined by Bates et al. (2015). Our modeling approach (following Barr et al., 2013) included random intercepts for subject and item to account for variability across subjects and items (PV or NP in sentence reading tasks; probe words). The maximal random effects structures incorporated in preliminary models were:

For PV or NP analysis: (1 + context + log(phraselength) + log(trialorder) | subject) + (1 + context * log(VST) | PV or NP item).

For probe word analysis: (1 + log(VST) + log(probelength) + log(probefrequency) + log(trialorder) | subject) + (1 + context * log(VST) | probe word item).

By-subject random slopes allow for differences among subjects in terms of their degree of sensitivity to phrase or word properties and treatment contexts; in contrast by-item random slope allows each experimental item to function differently depending on participant proficiency difference, treatment contexts and their interaction.

Linear mixed-effects models thus built analyzed the early and late sentence reading measures for both the PV and post-post noun phrase regions: first fixation duration, first-pass reading time, total reading time and second pass reading time data were examined. Independent variables of interest included context and proficiency indicated in term of VST. Phrase length and trial order were treated as covariates. The categorical variable ‘context’ was dummy-coded using neutral condition as the reference level. We were interested to look at context contrasts between neutral vs. figurative, neutral vs. literal, figurative vs. literal, and whether VST (proficiency) would interact with context in affecting eye fixation duration (log transformed). Instead of removing data prematurely, we kept all data points. For normality, all continuous variables were log-transformed to reduce skewing (expontentiating will convert the log values back to their original values). We used the *vif.mer* function to inspect multilinearity, *scale* to reduce collinearity and have all continuous predicting factors centered at their means.

Then we stepwise reduced the complexity of the random effects by removing from the maximal random structure the interactions (one at a time), random correlations (one at a time), random slopes (one at time) and/or random correlations until the models converges. Finally the most parsimonious models were selected using backward model selection based on anova () to compare models [see Ma et al. (2022)]. Since we kept all data points, the final models were refined through model-based residual analysis for outlier elimination (using *filter* (dataframe, abs (*scale* (resid (final. Model)) < 2.5 which means that any standardized residue with an absolute value greater than 2.5 is considered an outlier) (see Wu, 2019 for rationale). For fixed effects and interaction effects we relied on *mixed* in the *afex* package, and *emmeans* (Lenth et al., 2018) for the *post hoc* paired *t*-tests.

In the *glmer* mixed-effects models analyzing looks proportions of probe words, we initially included as fixed effects: visual probe word type (with figurative-unrelated control word as the reference level), context (with neutral condition as the reference level), VST indicating proficiency, and the covariates such as probe word frequency and length, and trial order. The categorical variables (context and probe word type) were dummy-coded. We were interested to look at context contrasts between neutral vs. figurative, neutral vs. literal, figurative vs. literal, and also probe type contrasts between figurative control vs. figurative related, literal control vs. literal related, figurative related vs. literal related, and whether VST (proficiency) interact with context and probe word type in affecting looks to the interest regions.

Since logistic regression is a statistical method to model the linear relationship between the predictor variables and log odds of a binary outcome such fixation and non-fixation (yes/no) events, the coefficient estimate (*β*) represents the change in log odds of the outcome per unit change in a predictor (between one categorical level and a reference level). Consequently, exponentiating these coefficiencts provides odds ratios which are easier to interpret as they translate log odds into probabilities or relative likelihood of fixation. An odds ratio greater than 1 suggests higher likelihood of fixation for one category over another of the predicator. With a log odds ratio of 0.21 for instance, exponentiating gives approximately 1.23, indicating that fixation to one categorical level is about 1.23 times (23% higher) the likelihood of fixating the reference level. Once one is no longer puzzled at the small log odds values of the coefficient estimate (*β*), it is suffice to rely on the log odds and the significant value to judge whether the outcome of fixation (looks) proportion change is significant as per change between a predictor level and its reference level.

We also abided by the *Keep it maximal* principle to build the random effect structure models, then stepwise reduced models until they converged, and selected the most parsimonious models (Barr et al., 2013). Results of the final best-fitting models for both sentence and probe reading were reported in the following section (alpha-levels were set at 0.05). Likewise, for normality, all continuous variables were log-transformed to reduce skewing. We used the *vif.mer* function to inspect multilinearity, *scale* to reduce collinearity and have all continuous predicting factors centered at their means. For fixed effects and interaction effects we utilized *mixed* in the *afex* package, and *emmeans* for the *post hoc* paired *t*-tests.

## Results

### Results from early and late measures at the sentence-reading stage

The table below provides descriptive statistics for early measures (first fixation duration and first pass reading time), as well as late measures (total reading time and second pass reading time) in the PV region and post-PV region across three context conditions (Table 1).

**Table 1:** Mean phrase-level early and late measures for PVs and post-PV NPs in context.

Interest region	Context	Early measures		Late measures	
		First fixation duration	First pass reading time	Total reading time	Second pass reading time
PV	Neutral	237.(123.)	399.(232.)	1,206.(700.)	806.(675.)
	Figurative	234.(92.8)	407.(227.)	1,029.(639.)***	622.(604.)***
	Literal	228.(91.6)	388.(223.)	1,056.(663.)***	669.(624.)***
Post-PV NP	Neutral	244.(96.4)	397.(206.)	1,115.(707.)	717.(667.)
	Figurative	238.(94.2)	415.(244.)	915.(586.)***	500.(546.)***
	Literal	248.(95.3)	413.(221.)	1,031.(637.)	619.(591.)

Duration measures are reported in milliseconds. Values in parentheses are SDs (standard deviations). Total reading time by definition excludes regressive fixations to preceding words, so second pass reading time = Total reading time - First pass reading time. In case of zero difference between total reading time and first pass reading time, 0 was converted to 1 so as not to affect log-transformation [see Wolter and Yamashita (2018)]. Significant differences between neutral baseline and treatment conditions (based on the models reported in Supplementary material C) are indicated with the convention of *p < 0.05, **p < 0.01, ***p < 0.001.

**Figure 3:**
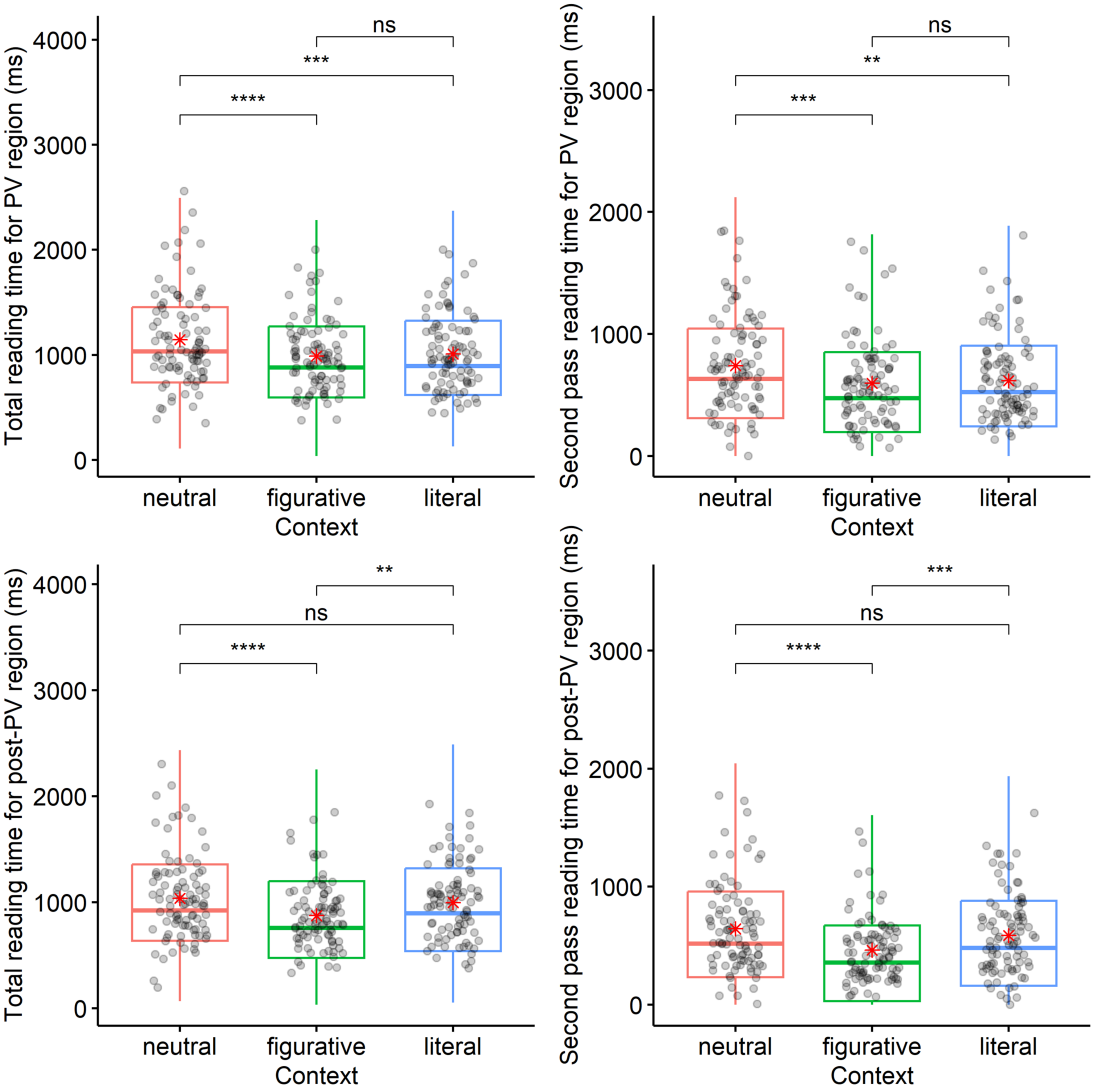
Late measures of durations (untransformed) for PV and post-PV noun phrase at sentence reading stage: *above left figure* shows total reading time for PV region in contexts; *above right figure* shows second pass reading time for PV region in contexts; *below left figure* shows total reading time for post-PV region in contexts; *below right figure* shows second pass reading time for post-PV region in contexts. In the *above* figures for the PV region, both total reading time and second pass reading measures have higher median lines and greater variability in the neutral context than in the figurative and literal contexts as indicated by the jittered points depicting individual participant averages. In the *below* figures for the post-PV region, both total reading time and second pass reading measures however have lower median lines and less variability in the figurative context than in the neutral and literal contexts. Besides, the red asterisks in the figures represent means values in each context for each measure. Significance levels in means comparison are indicated as follows: *p* < 0.05*, *p* < 0.01**, *p* < 0.001***, with “ns” denoting non-significant results. Refer to Supplementary material D for the 1st quartile, median, 3rd quartile for each of the four measures across contexts, and also relevant means values and standard deviations (SDs) as derived from model-based residual analysis to eliminate outliers.

In the best fit models^3^ (see Supplementary material C), the early measures on first fixation duration and first pass reading time showed no significant context main effects in both the PV region [*χ*^2^ (2) = 1.71, *p =* 0.424; χ^2^ (2) = 1.50, *p =* 0.473] and post-PV region [*χ*^2^ (2) = 2.71, *p =* 0.258; χ^2^ (2) = 1.42, *p =* 0.490], neither were there main effects of proficiency-indicating logged VST for the PV region [*χ*^2^ (1) = 0.77, *p =* 0.381; *χ*^2^ (1) = 0.66, *p =* 0.417] and for the post-PV region [χ^2^ (1) = 0.45, *p =* 0.500; χ^2^ (1) = 1.15, *p =* 0.284], or any interaction between the two factors. The late measures of total reading time and second pass reading time (Figure 3) showed no proficiency main effect in the two regions either [for the PV region: *χ*^2^ (1) = 0.66, *p =* 0.417; *χ*^2^ (1) = 0.32, *p =* 0.572; for the post-PV region: *χ*^2^ (1) = 0.30, *p =* 0.582; *χ*^2^ (1) = 0.61, *p =* 0.437]. However, more interestingly, in both regions the two late measures of log transformed fixation durations showed significant context main effect. In the PV region the main context effects for the total reading time and second pass reading time are as shown in the bracket [*χ*^2^ (2) = 23.90, *p <* 0.001; *χ*^2^ (2) = 15.29, *p <* 0.001]. Total reading time in the PV region was longer in the neutral context than in both figurative and literal contexts (neutral vs. Figurative: *β =* 0.1645, *t =* 4.83, *p =* 0.0002; neutral vs. Literal: *β =* 0.11, *t =* 3.17, *p =* 0.0002). Second pass reading time in the same region manifested the same pattern [neutral vs. Figurative: *β =* 0.18, *t =* 3.78, *p* < 0.0001; neutral vs. Literal: *β =* 0.13, *t =* 2.75, *p =* 0.006]. In the post-PV noun phrase region, the main context effects for total reading time [*χ*^2^ (2) = 35.23, *p <* 0.001] and second pass reading time [*χ*^2^ (2) = 26.69, *p <* 0.001] exhibited a different context contrast pattern (Figure 3). The figurative context resulted in shorter duration than neutral and literal contexts (for total reading time, neutral vs. figurative: *β =* 0.21, *t =* 5.78, *p* < 0.0001; figurative vs. literal: *β =* −0.15, *t =* −4.22, *p* < 0.0001; for second pass reading time, neutral vs. figurative: *β =* 0.27, *t =* 5.09, *p* < 0.0001; figurative vs. literal: *β =* −0.20, *t =* −3.64, *p =* 0.0003).

To sum up, early measures did not reveal difference in meaning activation between conditions along the proficiency range but late measures indicate more information reanalysis in neutral context than in literal and figurative contexts for the PV region. In the post-PV noun phrase region, for delayed PV processing, figurative meaning processing required less total reading time and second pass reading time, indicating the need of less information reanalysis in figurative context than in neutral and literal contexts.

### Results from further delayed visual word search task

**Figure 4:**
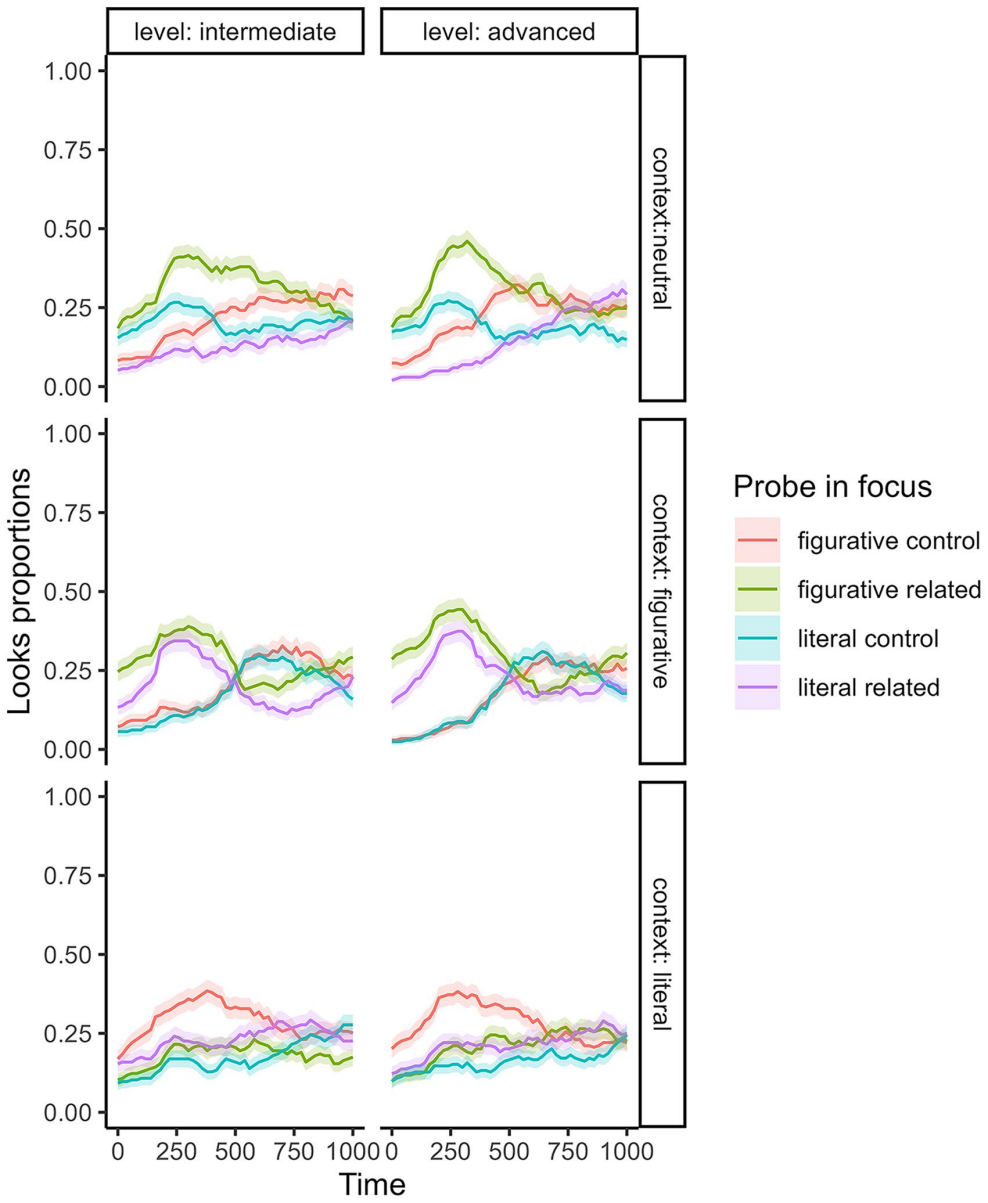
Probing word looks over a 1,000 ms time course in both proficiency groups: The figure displays curves showing proportions of looks to figurative control, figurative related, literal control, and literal related probe words within neutral, figurative, and literal contexts for intermediate and advanced participants.

The results below demonstrate further delayed PV processing effects after sentence reading. The looks proportions in neutral, literal, and figurative contexts across two proficiency levels are illustrated in Figure 4. The activation patterns within an initial 1,000-ms window were similar between intermediate and advanced groups.

### 180-580-ms T1 time window analysis

**Figure 5:**
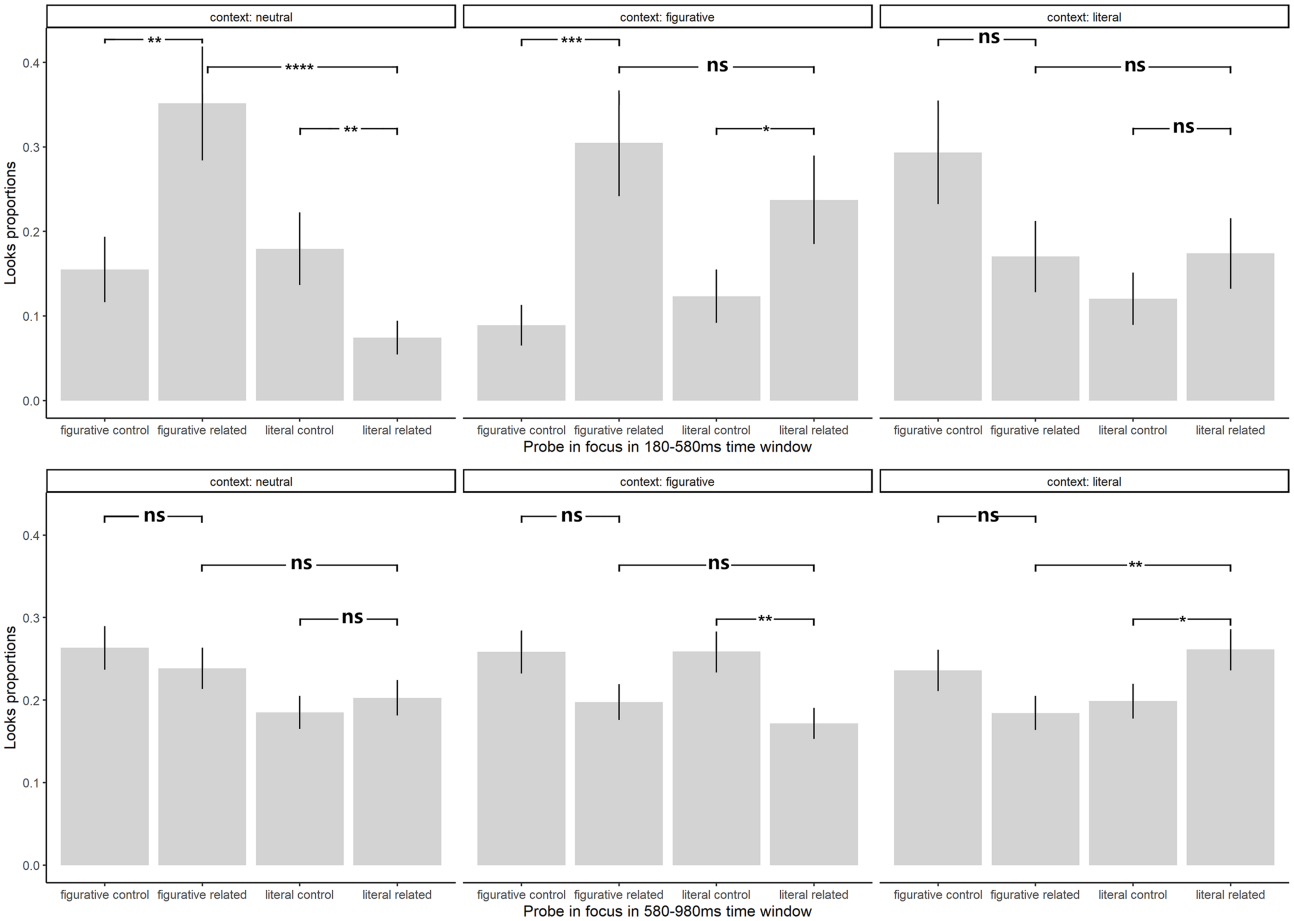
Probing word looks proportions across two time windows (180–580 ms and 580–980 ms): Significance levels of *post hoc* emmeans comparisons were determined using linear mixed effect models (*p* < 0.05*, *p* < 0.01**, *p* < 0.001***), with “ns” indicating non-significant results. Refer to Supplementary material D for detailed emmeans comparison data.

For the T1 window (Figure 5 *above*), the best fit model was Model: looks proportion ~ probe type in focus * context + log(VST) + log(order) + log(probe word length) + (1 | subject) + (1 | probe word item). Context and order showed main effects [*χ*^2^ (2) = 20.63, *p <* 0.001; χ^2^ (1) = 8.82, *p =* 0.003]. There was also interaction between probe word type and context [*χ*^2^ (6) = 3018.74, *p* < 0.001] *Post hoc* pairwise (literal related vs. literal control; figurative related vs. figurative control; literal related vs. figurative related) comparison confirmed that looks to the types of probe words focused varied depending on context.

In the neutral context there was significantly more looks to (consideration of) the figurative related probe (β_figurative related vs.literal related_ *=* 1.97, *z =* 4.59, *p* < 0.0001; β_figurative related vs. figurative control_ *=* 1.09, *z =* 2.68, *p =* 0.007) and the literal probe was less fixated and considered than its control word (β_literal related vs. literal control_ *= −*1.00, *z = −*2.60, *p =* 0.009).

In the figurative context, proportions of looks to the figurative probe were significantly higher than looks to the semantically unrelated control probes (β_figurative related vs. figurative control_ *=* 1.50, *z =* 3.67, *p =* 0.0002); the difference of looks to the literal and literal control probe was also at significance level (β_literal related vs. literal control_ *=* 0.80, *z =* 2.06, *p =* 0.040). But looks to the figurative and literal probes were comparable (β_figurative related vs. literal related_ *=* 0.40, *z =* 0.93, *p =* 0.353).

In the literal context, the most attention was given to the figurative control probe (β_figurative control vs. figurative related_ *=* 0.70, *z =* 1.72, *p =* 0.086). Looks to the literal probe were not much different from the figurative probe and the literal control probe (β_literal related vs. figurative related_ *= −*0.03, *z = −*0.06, *p =* 0.95; β_literal related vs. literal control_ *=* 0.43, *z =* 1.11, *p =* 0.269).

### 580-980-ms T2 time window analysis

For the T2 window (Figure 5 *below*), the best fit model was looks proportion ~ probe type in focus * context + log(VST) + log(order) + log(probe word length) + (1 | subject) + (1 | probe word item). There was main effect only with probe word length [*χ*^2^ (1) = 9.12, *p =* 0.003]. However there was an interaction between probe word type and context condition [*χ*^2^ (6) = 437.55, *p* < 0.001].

Post hoc comparison revealed that in the neutral context in the T2 window, proportions of looks to the figurative probe numerically remained higher (β_figurative related vs. literal related_ *=* 0.15, *z =* 0.78, *p =* 0.436). But the figurative control probe and literal related probe both competed for attention and consideration so that significance-level differences that had existed in the T1 window disappeared (β_figurative related vs. figurative control_ *= −*0.14, *z = −*0.75, *p =* 0.45; β_literal related vs. literal control_ *=* 0.12, *z =* 0.65, *p =* 0.516).

In the figurative context, more looks were now given to the control probe (β_figurative control vs. figurative related_ *=* 0.35, *z =* 1.91, *p =* 0.056; β_literal control vs. literal related_ *=* 0.52, *z =* 2.93, *p =* 0.003) while proportions of looks to the literal probe and figurative probe were both at a low (β_literal related vs. figurative related_ *= −*0.12, *z = −*0.59, *p =* 0.556).

In the literal context, the overall looks pattern remained similar to that in the T1 window, with least consideration of the literal control probe. Looks to the figurative control probe remained the highest but not significantly more than the figurative related probe (β_figurative control vs. figurative related_ *=* 0.32, *z =* 1.71, *p =* 0.088) but proportion of looks to the literal related probe increased to a significance level compared to the literal control probe and figurative related probe (β_literal related vs. literal control_ *=* 0.36, *z =* 2.03, *p =* 0.043; β_literal related vs. figurative related_ *=* 0.51, *z =* 2.60, *p =* 0.010).

## Discussion

The results do not support a simple ‘yes’ answer regarding whether figurative or literal meanings are preferentially activated across all contexts, nor does proficiency level necessarily modulate meaning activation patterns for familiar PVs.

Eye movements in sentence reading revealed no difference in early meaning activation across contexts. However, late meaning activation was observed in supporting literal and figurative contexts, along with delayed post-PV figurative meaning activation preference. There was no main effect from proficiency or any interaction effects.

The results from sentence reading indicate that early processes such as familiarity checks, access to orthographical/phonological information, lexical meaning retrieval, and early integration of information demand comparable cognitive effort in both neutral and biased contexts. As measured by first fixation duration and first pass reading time in both PV and post-PV regions, the preference for meaning activation was not discernible at this stage.

The late measure of total reading time for the PV region indicates that contextually biased meanings are more accessible in figurative and literal contexts compared to neutral ones. The other late measure—second pass reading time analysis—confirms that significantly more cognitive effort is required to perform information reanalysis in unbiased, neutral context than in the biased contexts. Thus learners can access familiar figurative and literal meanings with equal ease as indicated by the late measures, similar to what Siyanova-Chanturia et al. (2011) find about idiom processing by native speakers.

Further examination of post-PV spilloff region data shows that delayed information reanalysis may be less demanding in figurative contexts compared to other conditions. Since activation of preferred meaning is more likely to occur in supporting prior contexts, while activation of less expected meanings are likely to be suppressed despite boost from their supporting prior contexts (Holsinger and Kaiser, 2013), figurative meaning preference in figurative context together with no literal meaning activation in literal context may mean more salience of the figurative meanings for familiar PVs, though this preference emerges late in the post-PV spilloff region. Moreover, since no significant interaction effect was observed between proficiency and context on meaning activation across all models, this late figurative preference may be consistent across different proficiency groups.

The early measures used in sentence reading did not distinguish meaning activation preference across different contexts. However, since these early measures involve accessing word information and lexical meaning, the observation of no context effect at the early PV processing stage does not preclude the activation of literal or figurative meaning per se. Evidence for early literal or figurative activation was found in other studies (Holsinger, 2013; Kessler et al., 2020; Paulmann et al., 2015). For instance, Holsinger (2013) reported early literal activation during L1 idiom processing in a neutral context. Similarly, Kessler et al.'s (2020) empirical research confirmed early literal activation for idioms among L1 speakers within a neutral context; however, this effect disappeared shortly after the auditory cue stimulus offset. In contrast, Paulmann et al. (2015) found that even non-native English L2 speakers interpret PVs figuratively in neutral contexts like native English speakers, as evidenced by their ERP N400 measures. Future research remains necessary to determine if L2 literal activation is obligatory at an early stage.

In our read-only visual word search task at the further delayed stage, participants did not show explicit advantage or significant preference for literal meaning activation in neutral contexts. Additionally, they displayed no notable preference for activated literal meanings in figuratively biased contexts. However, there was a very late T2-time-window-specific preference for the literal meaning observed in literally biased contexts. These findings only partially support the literal salience hypothesis, which suggests that literal processing is obligatory even 800 ms after sentence offset. Instead, our results imply that context plays a crucial role in determining whether literal or figurative activation preference occurs at such further delayed stages of processing. In neutral contexts, participants preferred figurative meanings over literals during T1 time window. For comparison, the figurative context not only boosted figurative activation previously in the post-PV region when reading sentence, but also maintained figurative activation now in the T1 time window; meanwhile competing control words received more attention in T2 window and literal activation in the same context decreased from T1 to T2 time windows. In contrast, even within a literal context, delayed literal meaning activation occurred primarily in T2 window, suggesting that despite literal contextual boosts, literals were activated at the latest time.

Given our read-only visual word search task, which examined activation patterns 500 ms after sentence reading ceased, it’s plausible that any transient literal activation may have already subsided at this point. This aligns with Kessler et al.'s (2020) findings from their contrast of listen-and-look and read-only tasks in neutral contexts. Their experiments revealed no evidence of literal activation in the read-only visual word search task, suggesting that brief literal activation may not persist beyond the sentence reading stage. This finding supports our observation in the neutral context at this deferred stage in visual word search task.

Our results challenge Wang *et al*.’s (2016) finding that familiar PVs’ literal meanings were activated consistently regardless of whether the context was literal or figurative in the visual word search task 500 ms after sentence reading. Additionally, only advanced learners in their study exhibited activation for figurative meaning in literally biased contexts. Their interpretation suggested that learners at intermediate and advanced levels primarily relied on literal meanings in online PV processing. Their finding can be attributed to two limitations: their use of inappropriate statistical analysis and misinterpretation of results from their look-only visual word search task for early preference of literal meanings as clarified in the review section.

Wang et al.’s methodology involved proportions analysis of reading times for probe words over a longer trial duration (4,000 ms), whereas our study focused on shorter time frames within 1,000 ms typical for visual word search experiments. To further explore the processes, we also analyzed earlier-stage sentence reading as suggested by Reviewer One. These additional analyses provide complementary insights into early stages of PV meaning activation in our experimental tasks.

Notably, there was no observed proficiency effect for intermediate and advanced learners of English as a foreign language, suggesting that even lower-proficiency participants could activate familiar opaque PV meanings. Learner familiarity with PVs is likely to be more responsible for the facilitated access and retrieval of their figurative meanings. This finding aligns with Zhou and Zhang's (2011) research on idiom processing in neutral and figuratively biased contexts with two different levels of learners, which we now extend to PVs. To move one step further, by analyzing learners’ eye movements in PV and post-PV regions when they read context sentences as well as looks proportions on probe words in visual word search task, we pinpointed when literal and figurative activation may occur in context by both intermediate and advanced learners of English.

Our findings suggest that familiarity plays an important role in driving meaning activation, potentially outweighing proficiency levels among learners of English as a foreign language. When there is controll for the learner’s familiarity with both literal and figurative meanings of target PVs, differences in intermediate versus advanced L2 proficiency may not emerge. However, since this study focused solely on familiar PVs, conclusions about proficiency derived from these findings may not necessarily apply to unfamiliar ones.

This observation contrasts with previous studies where proficiency effects were observed: Matlock and Heredia (2002), Blais and Gonnermn (2013), Paulmann et al. (2015), and Wang et al. (2016). The findings from such studies might have been influenced by the unknown status of figurative language among lower-proficiency groups, as those previous studies did not assess learners’ familiarity with figurative meanings. For instance, in Matlock and Heredia's (2002) study on late bilinguals processing PVs literally rather than figuratively potentially due to their lack of awareness about figurative meanings. Similarly, Blais and Gonnermn (2013) and Paulmann et al. (2015) studies may have relied on higher-proficiency speakers’ knowledge of PVs’ figurative meanings, whereas lower-proficiency learners processed PVs literally because they were unfamiliar with the figurative meanings—no translation or paraphrasing tasks ensured such knowledge.

### Limitations

Sample size and generalizability: While the sample of 92 valid participants together with the current PV item sample provided sufficient statistical power of 0.8 calculated by G*Power, increasing the number of PVs tested could further enhance generalizability.

Focus on familiar PVs: Our investigation was limited to familiar PVs with equivalent familiarity ratings for both literal and figurative meanings. It remains uncertain how unfamiliar PVs are processed in context, as previous studies have preliminarily shown that familiar and unfamiliar idioms are processed differently across proficiency levels in biased contexts (Giora, 1999; Zhou and Zhang, 2011). Future research should explore the processing of unfamiliar PVs.

Literal activation patterns: Our findings regarding limited literal activation were observed within a specific 1,000-ms time frame—500 ms after sentence reading—and in earlier sentence reading there was no evidence to draw definitive conclusions about whether early literal or figurative activation was stronger in the current design. We would like to suggest a listen-and-look visual word search task to tap into early meaning activation directly for future research. Alternatively the parafoveal processing paradigm, as suggested by Reviewer One, may also provide direct evidence of real-time PV processing (Rayner, 1975; Schotter et al., 2015).

To advance our understanding of PV processing in L2 learners, we recommend the following: (1) increasing the sample size of experimental items; (2) investigating both familiar and unfamiliar PVs; and (3) observing patterns of meaning activation at different time points in the above-suggested paradigms.

## Conclusion

Though Cieslicka (2011) literal salience resonant hypothesis cannot be rejected based on current evidence with PVs, there is good evidence against claiming an obligatory preference for literal meanings regardless of context. Early measures from our study did not support either early literal or figurative meaning activation preference, suggesting that preference for literal versus figurative meanings may not emerge until later stages of processing. Late measures on the PV region indicate that both familiar literal and figurative meaning PVs enjoy similar ease of handling across contexts and late measures on the post-PV noun phrase region suggest that there is delay before figurative activation preference develops. In the visual word search task, no stable reliance on literal meaning was evident over time windows or contexts, further supporting the nuanced nature of PV processing beyond mere preference for literal meanings alone.

The findings also suggest that Giora (2002) graded salience hypothesis does not fully apply to this type of PVs with similar familiarity ratings for their literal and figurative meanings. Figurative activation is context-specific and time-sensitive, even among familiar PVs.

Last but not least, the findings underscore the importance of controlling for PV familiarity when investigating L2 learners’ comprehension across different proficiency levels. To further clarify early literal activation preference and proficiency effect in L2 learners’ PV processing mechanisms, additional research into both familiar and unfamiliar PVs in one framework among L2 learners across a broader range of proficiency using even more sensitive research paradigms would be beneficial.

## Data Availability

The original contributions presented in the study are included in the article/Supplementary material, further inquiries can be directed to the first author (yujianyao@scnu.edu.cn) or corresponding author (blacrose@163.com; blacrosezhang@suda.edu.cn).

## Footnotes

^1^Launching eye movement usually takes about 180 ms, and therefore the first 180-ms sampling data cannot be considered as valid data (Matin et al., 1993; Altmann and Kamide, 1999).

^2^TEM 4 and 8 are national proficiency tests for English Majors in China, and international proficiency level alignment research from China Ministry of Education shows that students who pass the tests are at intermediate and advanced English levels (Liu and Yang, 2021).

^3^The most parsimonious best-fitting models retained by-subject and by-item random intercepts for all outcomes except the first fixation duration model in the PV region. Due to issues related to near-zero variance associated with including a term for PV item-level random effect, its final model (Model: log(first fixation duration) ~ 1 + Context + log(PV length) + log(order) + log(VST) + (1 | subject)) only includes by-subject intercept.
