# Supplementary material for: The processing of familiar English L2 phrasal verbs in neutral and biased sentence contexts
Source: Front Psychol. 2025 Jun 5;16:1528821. doi: 10.3389/fpsyg.2025.1528821 (PMC12178310; doi:10.3389/fpsyg.2025.1528821)
Supplement: Supplementary file 1 [file Supplementary_file_1.docx]

Supplementary A.

# Target Phrasal Verbs and Probe Words in the Visual Display

| Phrasal Verbs | Literal related | Literal unrelated | Figurative related | Figurative unrelated |
| --- | --- | --- | --- | --- |
| stand by | lean | lend | support | search |
| run into | enter | exist | meet | move |
| come upon | near | name | encounter | embarrass |
| run across | rush | rest | discover | deliver |
| look into | see | show | check | catch |
| put out | take | tell | extinguish | endeavor |
| set down | place | prove | write | walk |
| blow up | enlarge | embark | explode | enforce |
| run after | chase | cheat | court | chill |
| come across | shortcut | scratch | find | feel |
| turn to | face | fill | approach | acquire |
| go through | cross | count | examine | express |
| call for | dial | dine | demand | defend |
