# Supplementary material for: The processing of familiar English L2 phrasal verbs in neutral and biased sentence contexts
Source: Front Psychol. 2025 Jun 5;16:1528821. doi: 10.3389/fpsyg.2025.1528821 (PMC12178310; doi:10.3389/fpsyg.2025.1528821)
Supplement: Supplementary file 2 [file Supplementary_file_2.doc]

# Supplementary B

# Material Sentences in the Experiment

| list | context | sentence |
| --- | --- | --- |
| 1 | neutral | He couldn't wait for any longer, so Danny blew up the balloons by himself. |
| 1 | neutral | Soon after he understood the situation, he called for a solution in no time. |
| 1 | neutral | While she was daydreaming, Sarah came across a castle casually. |
| 1 | neutral | When she was thinking hard about it, Anna came upon the man with a book in hand. |
| 1 | figurative | The police needed to search for clues of murder, so Tom went through the area with care. |
| 1 | figurative | As he wondered why the camera didn't work, Carl looked into the camera with care. |
| 1 | figurative | Being afraid of burning the house, Richard put out the candle before sleep. |
| 1 | figurative | When she was watching birds in the park, Eva ran across the house with big windows. |
| 1 | literal | Frank wanted to see his deskmake Jane play tricks on peers, so he ran after the girl for days. |
| 1 | literal | Sally joined the mad Black Friday shopping, and She ran into Zara on Oxford Street. |
| 1 | literal | To free hands and tie her shoe laces, Alice set down her list of shopping on the floor. |
| 1 | literal | As there weren't any empty seats on the bus, Jack stood by his brother on the way home. |
| 1 | literal | When Anna shared candy from the front seat, she turned to her sister first. |
| 2 | literal | He didn't have any pump at hand, so Danny blew up the balloons by himself. |
| 2 | literal | Once Jack found the number in the phone book, he called for a solution at once. |
| 2 | literal | As she wanted to reach the garden faster, Sarah came across a castle in her way. |
| 2 | literal | When she saw her boyfriend waiting at the gate, Anna came upon the man with joy. |
| 2 | neutral | As he knew it far better than anyone else, Tom went through the area with ease. |
| 2 | neutral | After everything else was done, Carl looked into the camera without anxiety. |
| 2 | neutral | As he changed his mind about the plan, Richard put out the candle right away. |
| 2 | neutral | Who knew what would happen to her next. Eva ran across the house in excitement. |
| 2 | figurative | Emma was charming and shining, so Frank ran after the girl for a long time. |
| 2 | figurative | Sally hadn't seen Zara for some time, but Sally ran into Zara on her way home today. |
| 2 | figurative | To make sure she would get gifts, Alice set down her list of shopping to her mom. |
| 2 | figurative | As they had promised to care for each other, Jack stood by his brother in all his life. |
| 2 | figurative | When Anna had problems to cope with, she turned to her sister often right away. |
| 3 | figurative | The party was over, so Danny used a pin and blew up the balloons very quickly. |
| 3 | figurative | The teacher was angry at campus bullying, so he called for a solution in no time. |
| 3 | figurative | As she turned her watchful eye down the street, Sarah came across a castle for sale. |
| 3 | figurative | When she was on patrol in the forest, Anna came upon the man half frozen. |
| 3 | literal | On Christmas people marched in the streets; Tom went through the area with joy. |
| 3 | literal | As he was curious about what it was, Carl looked into the camera once in a while. |
| 3 | literal | When he found his bag was too heavy, Richard put out the candle among others. |
| 3 | literal | When she saw the fire burning on the bed, Eva ran across the house for water. |
| 3 | neutral | It happened in middle school days. Frank ran after the girl for days. |
| 3 | neutral | It was vacation time at last. Sally ran into Zara on Oxford Street. |
| 3 | neutral | She was in a great hurry, so Alice set down her list of shopping before leaving. |
| 3 | neutral | They were twin brothers, so Jack stood by his brother quite a lot. |
| 3 | neutral | Nothing made the parents happier. She turned to her sister first among all things. |
