# Supplementary material for: The processing of familiar English L2 phrasal verbs in neutral and biased sentence contexts
Source: Front Psychol. 2025 Jun 5;16:1528821. doi: 10.3389/fpsyg.2025.1528821 (PMC12178310; doi:10.3389/fpsyg.2025.1528821)
Supplement: Supplementary file 3 [file Supplementary_file_3.docx]

Supplementary C

Best fit models

Sentence reading stage:

In the PV region

Model: log(first fixation duration) ~ 1 + Context + log(PV length) + log(order) + log(VST) +

Model: +(1 | subject)

Df full model: 8

Effect df Chisq p.value

1 Context 2 1.71 .424

2 log(PV length) 1 0.43 .512

3 log(order) 1 3.06 + .080

4 log(VST) 1 0.77 .381

Model: log(first pass reading time) ~ 1 + Context + log(VST) + log(PV length) +

Model: log(order) + (1 | subject) + (1 | PV item)

Df full model: 9

Effect df Chisq p.value

1 Context 2 1.50 .473

2 log(VST) 1 0.66 .417

3 log(PV length) 1 18.09 *** <.001

4 log(order) 1 6.73 ** .010

Model: log(total reading time) ~ 1 + Context + log(VST) + log(PV length) + log(order) +

Model: (1 | subject) + (1 | PV item)

Df full model: 9

Effect df Chisq p.value

1 Context 2 23.90 *** <.001

2 log(VST) 1 0.19 .662

3 log(PV length) 1 0.96 .326

4 log(order) 1 0.13 .717

contrast(emms1,interaction = "pairwise")

Context_pairwise estimate SE df t.ratio p.value

neutral - figurative 0.1645 0.0341 1070 4.831 <.0001

neutral - literal 0.1081 0.0341 1070 3.167 0.0016

figurative - literal -0.0564 0.0340 1070 -1.656 0.0980

Model: log(second pass reading time) ~ 1 + Context + log(VST) + log(PV length) +

Model: log(order) + (1 | subject) + (1 | PV item)

Df full model: 9

Effect df Chisq p.value

1 Context 2 15.29 *** <.001

2 log(VST) 1 0.32 .574

3 log(PV length) 1 0.29 .587

4 log(order) 1 0.20 .654

contrast(emms1,interaction = "pairwise")

Context_pairwise estimate SE df t.ratio p.value

neutral - figurative 0.1809 0.0479 921 3.780 0.0002

neutral - literal 0.1299 0.0472 922 2.754 0.0060

figurative - literal -0.0511 0.0482 922 -1.059 0.2899

In the post-PV NP region

Model: log(first fixation duration)~ 1 + Context + log(VST) + log(NP length) + log(order) +

Model: (1 | subject) + (1 | NP item)

Df full model: 9

Effect df Chisq p.value

1 Context 2 2.71 .258

2 log(VST) 1 0.45 .500

3 log(NP length) 1 0.09 .759

4 log(order) 1 0.04 .842

Model: log(first pass reading time) ~ 1 + Context + log(VST) + log(NP length) +

Model: log(order) + (1 | subject) + (1 | NP item)

Df full model: 9

Effect df Chisq p.value

1 Context 2 1.42 .490

2 log(VST) 1 1.15 .284

3 log(NP length) 1 18.96 *** <.001

4 log(order) 1 2.42 .119

Model:log(total reading time) ~ 1 + Context + log(VST) + log(NP length) + log(order) +

Model: (1 | subject) + (1 | NP item)

Df full model: 9

Effect df Chisq p.value

1 Context 2 35.23 *** <.001

2 log(VST) 1 0.30 .582

3 log(NP length) 1 4.79 * .029

4 log(order) 1 2.69 .101

contrast(emms1,interaction = "pairwise")

Context_pairwise estimate SE df t.ratio p.value

neutral - figurative 0.2051 0.0355 1068 5.781 <.0001

neutral - literal 0.0558 0.0353 1068 1.579 0.1147

figurative - literal -0.1494 0.0354 1068 -4.218 <.0001

Model: log(second pass reading time) ~ 1 + Context + log(VST) + log(NP length) +

Model: log(order) + (1 | subject) + (1 | NP item)

Df full model: 9

Effect df Chisq p.value

1 Context 2 26.69 *** <.001

2 log(VST) 1 0.61 .437

3 log(NP length) 1 3.41 + .065

4 log(order) 1 0.62 .432

contrast(emms1,interaction = "pairwise")

Context_pairwise estimate SE df t.ratio p.value

neutral - figurative 0.2680 0.0526 857 5.091 <.0001

neutral - literal 0.0724 0.0515 858 1.406 0.1601

figurative - literal -0.1956 0.0537 862 -3.642 0.0003

Visual word search stage:

180-580 ms

Model: looks proportion ~ probe type in focus * context + log(VST) + log(probe length) +

Model: log(order) + (1 | subject) + (1 | probe word item)

Data: data.analysis1

Df full model: 17

Effect df Chisq p.value

1 probe type in focus 3 4.76 .190

2 context 2 20.63 *** <.001

3 log(VST) 1 1.07 .301

4 log(probe length) 1 0.01 .930

5 log(order) 1 8.82 ** .003

6 probe type in focus:context 6 3018.74 *** <.001

emms1 <- emmeans(model1a,~probe type in focus|context,data=data.analysis1)

> contrast(emms1,interaction = "pairwise")

context = neutral:

probe type in focus pairwise estimate SE df z.ratio p.value

figurative control - figurative related -1.0937 0.409 Inf -2.677 0.0074

figurative control - literal control -0.1309 0.422 Inf -0.310 0.7567

figurative control - literal related 0.8740 0.427 Inf 2.048 0.0406

figurative related - literal control 0.9628 0.424 Inf 2.272 0.0231

figurative related - literal related 1.9676 0.429 Inf 4.585 <.0001

literal control - literal related 1.0049 0.387 Inf 2.596 0.0094

context = figurative:

probe type in focus pairwise estimate SE df z.ratio p.value

figurative control - figurative related -1.5015 0.409 Inf -3.670 0.0002

figurative control - literal control -0.3083 0.423 Inf -0.728 0.4664

figurative control - literal related -1.1037 0.426 Inf -2.588 0.0096

figurative related - literal control 1.1932 0.424 Inf 2.814 0.0049

figurative related - literal related 0.3978 0.428 Inf 0.929 0.3530

literal control - literal related -0.7954 0.386 Inf -2.059 0.0395

context = literal:

probe type in focus pairwise estimate SE df z.ratio p.value

figurative control - figurative related 0.7011 0.408 Inf 1.717 0.0860

figurative control - literal control 1.1560 0.422 Inf 2.737 0.0062

figurative control - literal related 0.7287 0.426 Inf 1.712 0.0868

figurative related - literal control 0.4548 0.424 Inf 1.072 0.2837

figurative related - literal related 0.0276 0.429 Inf 0.064 0.9487

literal control - literal related -0.4273 0.387 Inf -1.105 0.2690

580-980 ms

Model: looks proportion ~ probe type in focus * context + log(VST) + log(probe length) +

Model: log(order) + (1 | subject) + (1 | probe word item)

Data: data.analysis2

Df full model: 17

Effect df Chisq p.value

1 probe type in focus 3 2.24 .524

2 context 2 0.56 .756

3 log(VST) 1 1.32 .251

4 log(probe length) 1 9.12 ** .003

5 log(order) 1 3.00 + .083

6 probe type in focus:context 6 437.55 *** <.001

emms1 <- emmeans(m2,~probe type in focus|context,data=data.analysis2) ####

> contrast(emms1,interaction = "pairwise")

context = neutral:

probe type in focus pairwise estimate SE df z.ratio p.value

figurative control - figurative related 0.139 0.185 Inf 0.750 0.4531

figurative control - literal control 0.405 0.192 Inf 2.110 0.0348

figurative control - literal related 0.290 0.193 Inf 1.508 0.1315

figurative related - literal control 0.267 0.195 Inf 1.371 0.1705

figurative related - literal related 0.152 0.195 Inf 0.779 0.4360

literal control - literal related -0.115 0.177 Inf -0.649 0.5160

context = figurative:

probe type in focus pairwise estimate SE df z.ratio p.value

figurative control - figurative related 0.353 0.185 Inf 1.908 0.0564

figurative control - literal control -0.050 0.192 Inf -0.261 0.7944

figurative control - literal related 0.468 0.193 Inf 2.430 0.0151

figurative related - literal control -0.403 0.194 Inf -2.074 0.0381

figurative related - literal related 0.115 0.195 Inf 0.590 0.5555

literal control - literal related 0.518 0.177 Inf 2.931 0.0034

context = literal:

probe type in focus pairwise estimate SE df z.ratio p.value

figurative control - figurative related 0.316 0.185 Inf 1.706 0.0880

figurative control - literal control 0.169 0.192 Inf 0.877 0.3802

figurative control - literal related -0.189 0.192 Inf -0.983 0.3257

figurative related - literal control -0.148 0.195 Inf -0.757 0.4489

figurative related - literal related -0.505 0.195 Inf -2.595 0.0095

literal control - literal related -0.358 0.177 Inf -2.026 0.0428

**Note: all the continuous predicting factore were scaled and centralized.**
