# Supplementary material for: The processing of familiar English L2 phrasal verbs in neutral and biased sentence contexts
Source: Front Psychol. 2025 Jun 5;16:1528821. doi: 10.3389/fpsyg.2025.1528821 (PMC12178310; doi:10.3389/fpsyg.2025.1528821)
Supplement: Supplementary file 4 [file Supplementary_file_4.docx]

Supplementary D: Additional data for Figure 3

On Figure 3

about top left figure for total reading time for PV region in neutral, figurative and literal contexts:

1^st^ quartile _neutral_ = 737ms, median _neutral_ = 1032ms, 3^rd^ quartile _neutral_ = 1456ms;

1^st^ quartile _figurative_ = 594ms, median _figurative_ = 879ms, 3^rd^ quartile _figurative_ = 1270ms;

1^st^ quartile _literal_ = 618ms,median _literal_ = 893ms, 3^rd^ quartile _literal_ = 1324ms;

mean _neutral_ = 1145ms; mean _figurative_ = 988ms; mean _literal_ = 1008ms;

SD _neutral_ = 603ms; SD _figurative_ = 556ms; SD _literal_ = 549ms;

about top right figure for second pass reading time for PV region in neutral, figurative and literal contexts:

1^st^ quartile _neutral_ = 308ms, median _neutral_ = 630ms, 3^rd^ quartile _neutral_ = 1043ms;

1^st^ quartile _figurative_ = 194ms, median _figurative_ = 473ms, 3^rd^ quartile _figurative_ = 850ms;

1^st^ quartile _literal_ = 243ms, median _literal_ = 523ms, 3^rd^ quartile _literal_ = 903ms;

mean _neutral_ = 741ms; mean _figurative_ = 596ms; mean _literal_ = 617ms;

SD _neutral_ = 575ms; SD _figurative_ = 544ms; SD _literal_ = 505ms;

about bottom left figure for total reading time for post-PV region in neutral, figurative and literal contexts:

1^st^ quartile _neutral_ = 638ms, median _neutral_ = 924ms, 3^rd^ quartile _neutral_ = 1359ms;

1^st^ quartile _figurative_ = 477ms, median _figurative_ = 760ms, 3^rd^ quartile _figurative_ = 1199ms;

1^st^ quartile _literal_ = 541ms, median _literal_ = 896ms, 3^rd^ quartile _literal_ = 1322ms;

mean _neutral_ = 1037ms; mean _figurative_ = 875ms; mean _literal_ = 999ms;

SD _neutral_ = 567ms; SD _figurative_ = 506ms; SD _literal_ = 563ms;

about bottom right figure for second pass reading time in neutral, figurative and literal contexts:

1^st^ quartile _neutral_ = 234ms, median _neutral_ = 518ms, 3^rd^ quartile _neutral_ = 959ms;

1^st^ quartile _figurative_ = 31.5ms, median _figurative_ = 358ms, 3^rd^ quartile _figurative_ = 670ms;

1^st^ quartile _literal_ = 162ms, median _literal_ = 481ms, 3^rd^ quartile _literal_ = 879ms;

mean _neutral_ = 643ms; mean _figurative_ = 462ms; mean _literal_ = 587ms;

SD _neutral_ = 530ms; SD _figurative_ = 466ms; SD _literal_ = 525ms
